# The Cardioprotective Signaling Activity of Activated Protein C in Heart Failure and Ischemic Heart Diseases

**DOI:** 10.3390/ijms20071762

**Published:** 2019-04-10

**Authors:** Di Ren, Hemant Giri, Ji Li, Alireza R. Rezaie

**Affiliations:** 1 Department of Physiology and Biophysics, Mississippi Center for Heart Research, University of Mississippi Medical Center, Jackson, MS 39216, USA; dren@umc.edu; 2 Cardiovascular Biology Research Program, Oklahoma Medical Research Foundation, Oklahoma City, OK 73104, USA; hemant-giri@omrf.org; 3 University of Oklahoma Health Sciences Center, Oklahoma City, OK 73104, USA

**Keywords:** activated protein C, endothelial protein C receptor, heart failure, ischemic heart disease, cardioprotection

## Abstract

Activated protein C (APC) is a vitamin-K dependent plasma serine protease, which functions as a natural anticoagulant to downregulate thrombin generation in the clotting cascade. APC also modulates cellular homeostasis by exhibiting potent cytoprotective and anti-inflammatory signaling activities. The beneficial cytoprotective effects of APC have been extensively studied and confirmed in a number of preclinical disease and injury models including sepsis, type-1 diabetes and various ischemia/reperfusion diseases. It is now well-known that APC modulates downstream cell signaling networks and transcriptome profiles when it binds to the endothelial protein C receptor (EPCR) to activate protease-activated receptor 1 (PAR1) on various cell types. However, despite much progress, details of the downstream signaling mechanism of APC and its crosstalk with other signaling networks are far from being fully understood. In this review, we focus on the cardioprotective properties of APC in ischemic heart disease and heart failure with a special emphasis on recent discoveries related to the modulatory effect of APC on AMP-activated protein kinase (AMPK), PI3K/AKT, and mTORC1 signaling pathways. The cytoprotective properties of APC might provide a novel strategy for future therapies in cardiac diseases.

## 1. Protein C System

### 1.1. Protein C Zymogen and Its Activation Mechanism

Protein C (PC) is a vitamin K-dependent serine protease zymogen that is primarily synthesized by the liver and circulates in plasma with a concentration of 4–5 μg/mL [1]. The gene for PC is located on chromosome 2, encoding 9 exons with 419 amino acids in length [2]. The mature protein contains two chains linked together by a single disulfide bond between Cys141 and Cys277 [2]. The N-terminal light chain contains the gamma-carboxyglutamic acid (Gla) domain and two epidermal growth factor (EGF-1, EGF-2) domains while the C-terminal heavy chain contains the trypsin-like serine protease domain [3]. Protein C through its Gla-domain binds with a high affinity to endothelial protein C receptor (EPCR) and is converted to activated protein C (APC) through a limited proteolytic process by the thrombin-thrombomodulin (TM)- complex on the surface of endothelial cells [4,5,6]. TM is a single chain transmembrane type-1 receptor glycoprotein composed of an N-terminal lectin-like domain, six EGF-like domains, and a short cytoplasmic tail [7,8,9]. The EGF-5 and -6 domains of TM bind to thrombin to change the substrate specificity of thrombin from a procoagulant to an anticoagulant enzyme by rendering it inactive toward fibrinogen but enabling it highly active toward PC [4,10]. The EGF-4 domain of TM binds the PC to facilitate the presentation of the PC zymogen into the catalytic pocket of thrombin [10,11]. The Gla-domain of PC has a high-affinity binding site for EPCR and this interaction on the endothelial cell surface enhances the activation of PC by the thrombin-TM complex approximately 20-fold [6]. 

### 1.2. Anticoagulant Mechanism

Upon activation by the thrombin-TM complex, APC can dissociate from its membrane-bound receptor cofactor, EPCR, and bind to its circulating vitamin K-dependent plasma cofactor, protein S, on a negatively charged membrane surface, to initiate its anticoagulant function by proteolytically inactivating factors Va and VIIIa, the essential procoagulant cofactors for thrombin generation in both intrinsic and extrinsic pathways of the blot clotting cascade [12,13,14]. Inactivation of factor Va by APC is mediated through cleavages at three Arg306, Arg506, and Arg679 protease recognition sites [15,16]. The cleavage at the Arg306 site induces the dissociation of factor Va from the negatively charged membrane surface, thereby leading to the complete inactivation of the cofactor [17]. The APC-protein S complex proteolytically inactivates factor VIIIa by a similar manner through the cleavage of the cofactor at Arg336 and Arg562 recognition sites [18]. A defect in the protein C anticoagulant pathway is associated with a hypercoagulable state that increases the clotting propensity of the blood, thereby resulting in pathological vascular thrombosis with variable severity due to uncontrolled thrombin generation [19,20]. The classic example is a clinical-pathological condition referred to it as “APC resistance” which was used to describe the phenomenon of a poor response to the anticoagulant activity of APC [21]. It was later discovered that APC resistance is mainly due to a mutation in the APC’s target gene factor V in which Arg506 of the cofactor is replaced with a Gln [22]. The mutant cofactor was then named Factor V-Leiden which predisposes the carriers to a hypercoagulable phenotype due to the poor recognition and inactivation of the variant cofactor by APC [22]. APC resistance has been demonstrated as a cardiovascular risk factor for atherosclerosis and arterial thrombosis among patients with stroke and venous thrombosis [23,24].

### 1.3. Anti-Inflammatory and Cytoprotective Mechanism

**Figure 1:**
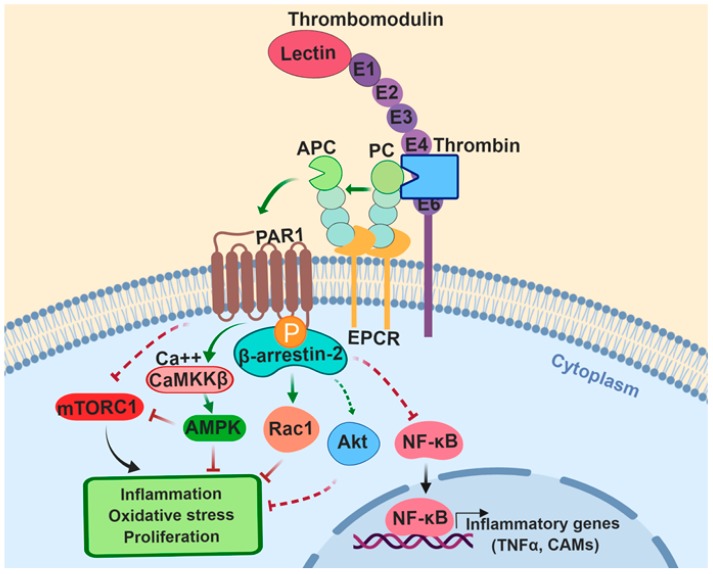
The protein C activation by the thrombin-thrombomodulin complex and the cytoprotective function of Activated Protein C (APC) in ischemic heart disease and heart failure. The Endothelial Protein C Receptor (EPCR)-dependent cleavage of Protease-Activated Receptor 1 (PAR1) by APC initiates β-arrestin-2 biased signaling that results in the activation of Rac1 GTPase, Akt, and AMPK by the Ca^2+^/calmodulin-dependent protein kinase-kinase β (CaMKKβ) pathway. The PAR1-biased signaling also inhibits the activation/nuclear translocation of NF-κB and mTORC1 signaling network. E1, E2… represent EGF-like domains. See the text for more details. The figure was prepared by software provided by Biorender.com.

In addition to functioning as an essential anticoagulant protease in the clotting cascade, APC also exhibits potent anti-inflammatory and cytoprotective signaling activities when it remains associated with EPCR on the membrane surface [25]. It has been demonstrated that the Gla-domain of PC and APC binds EPCR with a similar affinity [26]. The protective cellular signaling activity of APC is mediated through the EPCR-bound protease-activating protease-activated receptor 1 (PAR1) on endothelial or other cell types (Figure 1) [27]. The PAR1-dependent anti-inflammatory activity of the APC-EPCR complex mediates the inhibition of expression of inflammatory genes including the inhibition of key transcription factors like the activator protein 1 (AP-1) family c-Fos and FosB [28,29,30]. The protective signaling also results in inhibition of the release of inflammatory cytokines (such as IL-1β, IL-6 and TNF-α), the inhibition of the nuclear translocation of NF-κB and the down-regulation of endothelial cell adhesion molecules such as ICAM-1, VCAM-1 and E-selectin (Figure 1), thereby limiting the leukocyte infiltration through the vascular system [28,29,30]. PAR1 is a G-protein coupled receptor (GPCR) which was first identified as a specific receptor for thrombin on platelets [31]. However, later studies showed that other coagulation proteases can cleave the receptor through different recognition sites on the extracellular domain of the receptor to initiate distinct intracellular signaling responses [32,33,34]. Thrombin cleaves PAR1 at the Arg41 site of the receptor to elicit pro-inflammatory responses in endothelial cells by mediating the phosphorylation of ERK1/2 and activation of the RhoA signaling pathway, thereby leading to disruption of the endothelial barrier function and edema formation [25,35,36]. On the other hand, the APC-EPCR complex cleaves the Arg46 site of PAR1 to initiate anti-inflammatory and cytoprotective signaling responses that culminate in activation of Rac1 signaling that counteracts the barrier disruptive effect of RhoA signaling in vascular endothelial cells [34,36]. Interestingly, it has been found that the occupancy of EPCR by the Gla-domain of PC/APC plays a key role in determining the signaling specificity of PAR1 [34]. Thus, when EPCR is occupied by its natural ligand, the PAR1-dependent signaling specificity of coagulation proteases is cytoprotective independent of the proteases cleaving either Arg41 or Arg46 cleavage sites [37,38]. It has been demonstrated that the receptors of protein C activation (TM) and APC signaling (EPCR and PAR1) are all colocalized in lipid-rafts of endothelial cells, [39] and that the occupancy of EPCR by the Gla-domain of protein C recruits GPCR kinase-5 (GRK-5) to the membrane, thereby phosphorylating the cytoplasmic domain of PAR1 and favoring its interaction with β-arrestin-2 rather than with a G-protein (Figure 1) [38]. Thus, a β-arrestin-2 biased EPCR-dependent PAR1 signaling by either thrombin and APC initiates anti-inflammatory and cytoprotective and anti-apoptotic signaling responses in vascular endothelial cells [34,36,38].

The EPCR- and PAR1-dependent anti-apoptotic activity of APC is partially mediated through the down-regulation of expression of the typical intrinsic apoptosis pathway genes including p53 and Bax and up-regulation of the expression of the oppositely functioning gene Bcl-2 [40]. Additionally, APC has been found to counteract apoptosis by inhibiting caspase-8 activation, which is the typical extrinsic apoptosis pathway gene [41]. Moreover, APC was reported to regulate both the inflammation and apoptosis processes by regulating the endoplasmic reticulum calcium flux and reducing the reactive oxygen species (ROS) accumulation [42]. Even though the anti-apoptotic effects of the APC pathway has been confirmed both in vitro and in vivo systems, additional future studies are required to clarify the mechanism through which APC exerts anti-apoptotic effects under different pathophysiological conditions.

## 2. Ischemic Heart Disease and APC Cardioprotection 

### 2.1. Ischemia/Reperfusion (I/R) Injury and Cell Death

In the ischemic phase of heart disease, the ischemic injury is primarily caused by the blockage of the oxygen supply to heart tissues [43]. Insufficient oxygen supply results in a decrease in ATP synthesis by the mitochondria through oxidative phosphorylation. The energy and oxygen deficiency enhances the glycolytic rate and elevates H+ production, which eventually decreases the cytosolic pH. Meanwhile, the cytosolic Ca^2+^ channel is disturbed because the cell compensatory pH regulating system leads to an increase in Ca^2+^/Na^+^ exchange [43]. The accumulated Ca^2+^ would increase the plasma membrane permeability, increase the activity of cell-damaging proteases, and increase mitochondrial permeability by opening the mitochondrial permeability transition pore (mPTP). Such damage occurring in the mitochondrial membrane leads to the leakage of the electron transport chain and generation of ROS, primarily in the superoxide forms [43]. The oxidative stress is the main factor contributing to the intracellular injury that leads to necrotic cell death [43,44]. 

The re-entry of oxygen supply to myocytes is responsible for altering the intracellular metabolism and environment, thereby inducing further cellular damage called reperfusion injury [45,46]. The damage is mainly caused by the oxygen in the mitochondrial respiratory chain due to the activation and excessive activity of electron transferring enzymes leading to accumulation of ROS, oxidative stress and intracellular damage [47]. ROS is also involved in the activation of several pathogenetic networks including further increasing Ca^2+^ concentration, activating hydrolases and increasing mPTP that ultimately culminates in cell death [43,48,49]. Various signaling pathways have been reported to be significantly elevated or activated in the reperfusion phase, such as the mitogen-activated protein kinase family (MAPK), c-Jun N-terminal kinase (JNK), NF-κB, and apoptotic pathways [43,46,48,49]. The damaged cells from the I/R injury initiate inflammatory responses that result in the activation of macrophages, endothelial cells, neutrophils, lymphocytes, as well as the complement system, altering the expression levels of pro- and anti-inflammatory cytokines and inducing further cardiac cell death [43,44,45,46,47,48,49,50,51,52,53,54]. Three cell death mechanisms are involved in cardiac I/R injury. Apoptosis, a process of programmed cell death that is induced in I/R injury by the hypoxic stress and excessive oxidative stress from reperfusion [43]. It can be induced through the activation of the pro-apoptosis Bcl-2 family and other apoptotic protease-activating factors that compromise the integrity of the mitochondrial membrane, leading to caspase cascade activation and the degradation of mitochondria which is called mitoptosis [44,55,56]. Another type of programmed cell death, known as necroptosis in I/R injury could induce intensive local inflammation in ischemic tissue [56]. The key regulators of necroptosis are the RIP1 and RIP3 complex and the phosphorylated form of RIP3 can recruit RIP1 to activate the necrosome-induced necroptosis [57]. Additionally, the overexpression of RIP3 has been found to accompany excessive ROS generation and enhanced NF-κB transcription factor accumulation which is involved in the initiation of inflammation [58]. A compensatory cardioprotective signaling mechanism that is initiated in the stressed heart to cope with ischemic stress and ensuing energy depletion is the AMP-activated protein kinase (AMPK) signaling pathway [59,60,61,62]. The AMPK signaling can supply energy to the stressed heart through glycolysis and alternative metabolic pathways including autophagy through the process of degrading intracellular damaged organelles and macromolecules by lysosomal pathways [59,60,61,62,63,64,65]. In addition to providing ATP through alternative metabolic pathways, the AMPK signaling also exerts a cardioprotective effect in the I/R-induced stressed heart through inhibition of inflammatory MAPK and NF-kB signaling pathways [59].

### 2.2. Cardioprotective Effect of APC against I/R Injury

The observation that APC exhibits potent anticoagulant, anti-inflammatory and cytoprotective properties in conjunction with knowledge of the pathogenic mechanism of I/R injury which is associated with the up-regulation of pro-inflammatory and pro-apoptotic pathways as described above, providing the rationale for evaluating the cardioprotective function of APC in a couple of rat and mouse models of myocardial I/R injury [66,67]. The results indicated that treatment with APC during I/R restores the mean arterial pressure after a short time occlusion and reduces the mortality rate in experimental animals [66,67]. To understand the mechanism by which APC exerts a cardioprotective effect, we investigated this question by using human recombinant APC and its two signaling-selective and anticoagulant-selective derivatives in a mouse model of acute I/R injury [68]. The effect of APC derivatives was assessed on myocardial infarction size, post-ischemic cardiac function recovery and the modulation of inflammatory responses on cardiomyocytes [68]. We discovered that APC attenuates acute ischemic injury in the heart via stimulating the AMPK signaling and the inhibition of NF-κB and JNK signaling pathways by a mechanism(s) that is largely independent of its anticoagulant activity [68]. Thus, the administration of both APC and the signaling-selective APC-2Cys [69], but not the anticoagulant-selective and signaling-defective APC-E170A [70], reduced myocardial infarction and restored cardiac function in the ischemic mouse heart by activating AMPK in both in vivo and ex vivo model systems [68]. Further studies revealed that cardiomyocytes express EPCR and that both APC and APC-2Cys directly trigger AMPK phosphorylation in cardiomyocytes by enhancing the Ca^2+^/CaMKKβ activity by EPCR- and PAR1-dependent mechanisms (Figure 1) [68].

AMPK is a stress and energy sensitive kinase that can be activated by ATP depletion under ischemic stress [59,71]. AMPK is also an energy sensor and key regulator of metabolism mainly through glucose and fatty acid metabolism to maintain the homeostasis [59]. In myocardium, the AMPK-dependent activity of APC was found to stimulate glucose uptake through increasing the fusion of glucose transporter 4 (GLUT4) to the cell membrane and upregulating the glucose oxidation in the ischemic heart [72]. Interestingly, the upregulation of GLUT4 by the signaling-selective APC-2Cys variant was significantly higher than that of wild-type APC (72). In contrast to the upregulation of glucose uptake, APC-2Cys reduced fatty acid oxidation during I/R injury. This is an interesting observation since it is thought that the I/R-induced acceleration of cardiac fatty acid oxidation creates more ROS as compared to glucose oxidation. These results appear to suggest that APC-mediated reduction in fatty acid oxidation may lead to a decrease in ROS generation, thereby improving the intracellular redox status in the heart during I/R. Further support for this hypothesis was provided by the observation that APC-2Cys significantly increased the ratio of reduced glutathione (GSH) to oxidized glutathione (GSSG) in an ex vivo working heart perfusion system after 10 min of global ischemia and 20 min of reperfusion [72]. This method has been commonly used as an indicator of the intracellular redox status in the heart tissue.

Interestingly, both wild-type APC and the signaling-selective APC-2Cys also increased the autophagic flux in the heart following I/R [72]. This was demonstrated by measuring the LC3 II/LC3 I ratio as an indicator of autophagy during I/R, which was significantly enhanced with both the wild-type APC and APC-2Cys treatment groups [72]. The increase in the autophagic activity of APC is likely mediated through its activation of AMPK since it has been demonstrated that AMPK is involved in inducing autophagy in the heart during I/R [73]. The activity of APC-2Cys in modulating these metabolic pathways was significantly higher than wild-type APC during I/R since it uniquely enhanced glucose oxidation and attenuated the I/R-initiated fatty acid oxidation by 80% [72]. The mechanism by which the signaling-selective APC-2Cys variants exhibits these unique AMPK-dependent cardioprotective properties is not known and warrants further investigation. It is known that, in addition to EPCR and PAR-1, APC exerts its cytoprotective signaling activities through crosstalk with other G-protein (i.e., PAR-3 [74] and sphingosine 1-phosphate receptor 1 [25]) and a number of non-G-protein coupled (i.e., apolipoprotein E receptor 2 [75], Tie2 [76] and Mac1 [77]) receptors. Thus, it is possible that differences in interaction and/or crosstalk by APC and APC-2Cys with one or more of these receptors participate in their differential modulatory effect on substrate metabolism in the I/R-stressed heart. 

APC has been shown to induce the activation of AKT1 in the myocardium, thereby improving the endothelial function of the coronary artery in a global ischemic reperfusion rat model [78]. AKT is a serine/threonine kinase also known as protein kinase B (PKB) which activates mTOR, thereby playing multifaceted roles in the cellular metabolism, proliferation, inflammation, transcription and protein synthesis [79,80,81]. It has been recently found that the cytoprotective effects of APC in I/R injury are associated with the inhibition of mTORC1 signaling network, leading to inhibition of the NLRP3 inflammasome pathway [82]. It was demonstrated that the mTORC1 inhibitory effect of APC was mediated through APC, reducing the expression of the regulatory component of the mTORC1 complex (Raptor) [82], thereby inhibiting the phosphorylation of p70S6K, one of the downstream targets of the mTORC1 signaling pathway [83]. The mTROC1 signaling network is a target for down-regulation by the AMPK signaling pathway as well [59]. Thus, the exact cardioprotective mechanism of APC in the I/R injury and the possible elaborate and complex crosstalk between different signaling networks warrants further investigation. 

### 2.3. Cardioprotective Function of APC in Heart Failure

Mounting evidence in the literature assigns a key cardioprotective role for AMPK in various cardiovascular disease models [84]. It has been established that the activation of AMPK plays a protective role during the initiation and progression of heart failure (HF) by regulating the metabolism and maintaining the homeostasis [84,85,86,87,88]. The activity of AMPK has been found to regulate substrate metabolism/utilization in a rat model of pressure overload-induced cardiac hypertrophy, indicating a critical role for AMPK in cardiac adaptive response under pathological conditions [84]. It has been also found that the AMPKα2 activity protects the heart against pressure overload-induced HF through mediating estrogen-related receptor α (ERRα) using the AMPKα2 deficient mice [89]. In a recent study, both APC and the signaling-selective non-anticoagulant APC-2Cys were shown to protect against pressure overload-induced hypertrophy through the AMPK signaling pathway [90]. AMPK has been found to reduce ROS accumulation by inhibiting NADPH oxidase activation in various cardiac disease models including HF [91,92]. In the pressure overload model, the AMPK-dependent cytoprotective signaling function of APC was found to be critical in the inhibition of ROS accumulation and inflammation through the down-regulation of the activity of p66shc and expression of 4-HNE in the hypertrophic model [90]. Similar cytoprotective effects for APC have been reported in the diabetic nephropathy model through the protease down-regulating p66shc by a PAR1-dependent mechanism [93]. The cardioprotective function and mechanism of APC in HF patients requires further investigation. Whether the protective signaling function of APC is beneficial in HF models with preserved ejection fraction (HFpEF) or reduced ejection fraction (HFrEF) remains to be determined. 

In addition to its cardioprotective properties, APC exhibits potent cytoprotective and anti-inflammatory effects in a number of other acute and chronic diseases including sepsis [94], ischemic stroke [95], acute kidney injury [96], type-1 diabetes [97], wound healing [98], Plasmodium falciparum malaria [99], post-surgical adhesion band formation [100] and other inflammatory disorders that have been nicely reviewed in recent review articles [101,102]. There are several ongoing pre-clinical and clinical trials evaluating the potential therapeutic utility of APC in animals and humans [101,102]. The future studies with the signaling-selective APC derivatives which do not exhibit significant anticoagulant activities and thus are not associated with an increased risk of bleeding may provide APC-based therapeutic strategies for some of these inflammatory diseases.

## 3. Conclusions

Ischemic heart disease and heart failure are the leading causes of morbidity and mortality worldwide. There is an urgent need for developing new therapeutic strategies for heart diseases. Results of studies with APC in I/R and HF models are encouraging and warrant further investigation. Because of its potent anticoagulant activity, APC-therapy has been found to also be associated with an increased risk of bleeding. The findings that the signaling-selective APC, lacking anticoagulant function, has a similar cardioprotective function in the I/R injury and HF models may open a new avenue for further investigating the life-saving effects of APC in heart disease without increasing the risk of bleeding.
